# MicroCT data provide evidence correcting the previous misidentification of an Eocene amber beetle (Coleoptera, Cicindelidae) as an extant species

**DOI:** 10.1038/s41598-023-39158-7

**Published:** 2023-09-07

**Authors:** Joachim Schmidt, Stephan Scholz, Jürgen Wiesner, Kipling Will

**Affiliations:** 1 Institute of Biosciences, General and Systematic Zoology, University of Rostock, Rostock, Germany; 2 Zoologische Staatsammlung, Munich, Germany; 3 Essig Museum of Entomology, University of California, Berkeley, USA

**Keywords:** Entomology, Palaeontology, Phylogenetics, Taxonomy

## Abstract

The fossil record suggests some insect species have a marked longevity. The oldest fossils purported to represent extant insect species are from the Oligocene and Eocene. One of the most cited fossils is the extant tiger beetle *Tetracha carolina* (Coleoptera: Cicindelidae) that was identified over a century ago by Walther Horn in Eocene Baltic amber. We examined this and compared it to the previously described cincindelid Baltic amber fossil *Palaeoiresina cassolai* using X-ray microscopy and 3D imaging techniques. We conclude that Horn’s fossil tiger beetle specimen is conspecific with the Eocene *P. cassolai* and is a member of an extinct stem group lineage of Cicindelidae. Based on a review of all the tiger beetle fossils described from Cretaceous and Paleogene deposits, we found that the assignment of these fossil species to extant lineages is not supported. There are currently no synapomorphies known from fossils that can provide evidence for Cretaceous Manticorni or Megacephalini nor is there evidence for Eocene Iresina. We provide evidence that rejects the idea of a recent beetle species persisting since the Eocene period, which is crucial for using the currently known fossil Cicindelidae species to calibrate divergence dating of beetle phylogenies.

## Introduction

Unlike species of mammals, insect species are thought to have a marked longevity. Evidence for this comes from numerous instances where the fossil record shows that modern insect species were present during the Pliocene and Early Quaternary periods^1–3^. Though less common, some recent insect species have been identified from fossil deposits that are dated much earlier (see publication by Hörnschemeyer et al.^4^ for overview). The oldest fossil records of presumed recent insect species were reported from the lower Oligocene sedimentary rocks of the Florissant formation in the Rocky Mountains (~ 34 Ma^5^) and from Eocene Baltic amber (50–35 Ma^6^). However, for several reasons, these findings should be treated with great caution. Grimaldi and Engel^7^ hypothesize an average duration for insect species of ~ 3–10 My and that purported, significantly older records are the result of imprecise comparisons of the fossil and living specimens. Another problem is that the assignment of fossils to contemporary taxa may be based on general similarity rather than synapomorphies^8^. The set of morphological characters which can be obtained from fossil specimens is often too limited to make clear assignment possible. For example, this is the case for *Plateumaris primaeva* (Wickham) (Coleoptera: Chrysomelidae), a compression fossil preserved in Florissant shales, as shown by the phylogenetic study of the group by Askevold^9^. The available characters in this fossil are similar to those in the extant *P. nitida* (Germar) but the identity of the two taxa cannot be established as critical, species-distinguishing features of the male genitalia are not evident^9^. Therefore, including this fossil as an example of a recent species that survived since the Oligocene would be misleading^4,7^ despite the apparent stasis of visible features.

**Figure 1:**
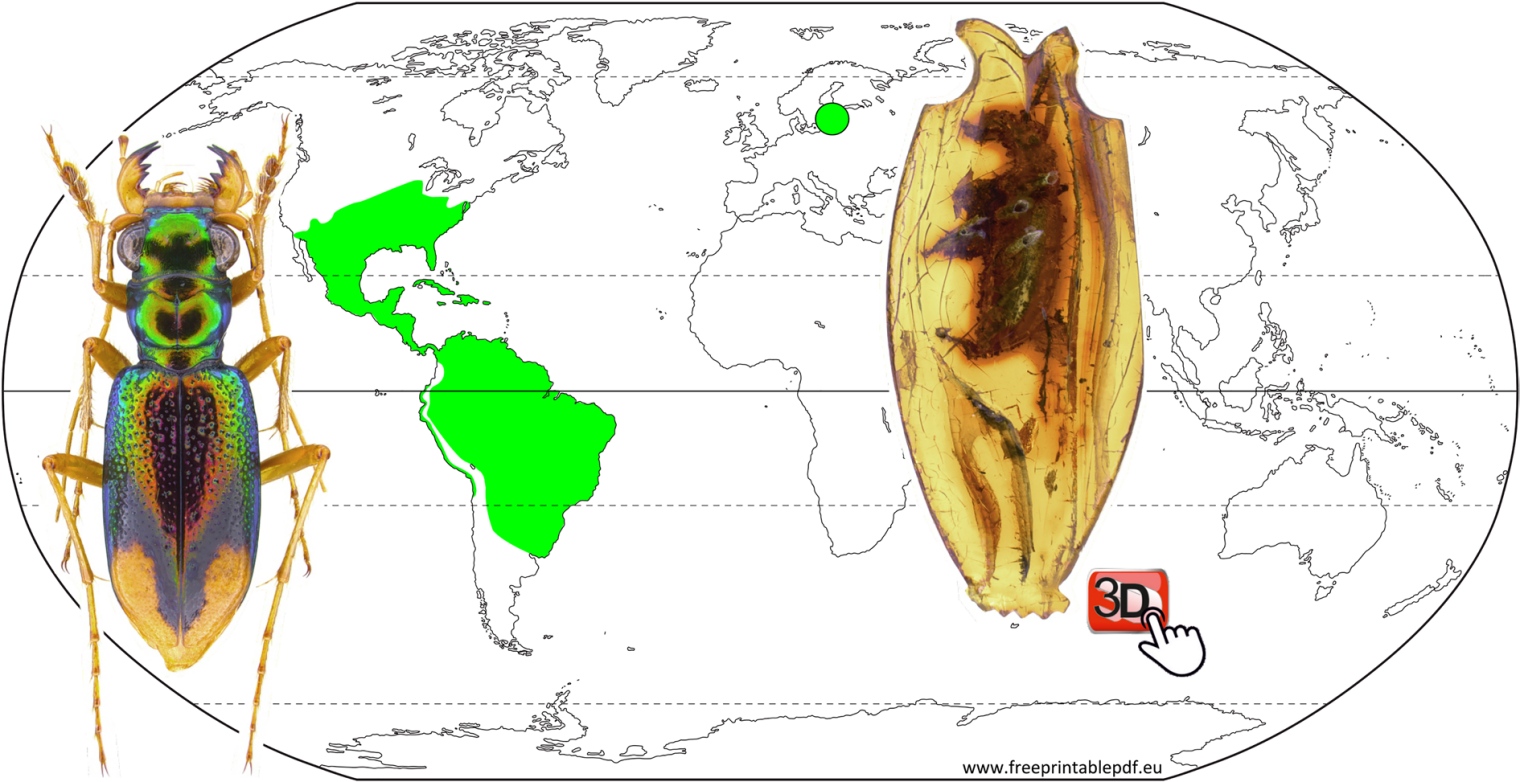
World map showing the current distribution of species of the genus *Tetracha* (green region) and the source locality of Horn’s tiger beetle fossil in the Kaliningrad region (green circle). The included images are of a specimen of *Tetracha carolina carolina* (left), the somewhat oddly cut piece of amber with the cicindelid fossil inclusion (right). The interactive PDF of the microCT reconstruction of Horn’s tiger beetle fossil as an embedded 3D image is available at Supplementary figure 1.

Significant technical advances in the study of fossils have led to the revision of previous views regarding the taxonomy of insect species. For example, the recent Palearctic *Nemadus colonoides* (Kraatz) (Coleoptera: Leiodidae) was identified by Jeannel^10^ based on a fossil specimen preserved in Baltic amber. However, MicroCT scanning made possible the study of the male and female genital characters of the fossils and led to the description of an Eocene species different from *N. colonoides*^11^. A more well-known fossil and example of a putative, long-lived insect species that was considered to have persisted since the Late Paleogene period is the tiger beetle *Tetracha carolina* (Linnaeus, 1763) of the Cicindelidae tribe Megacephalini^12–16^. The species was identified more than a century ago by Walther Horn^17^ from a fossil specimen embedded in a piece of Baltic amber from the Kaliningrad region. With about 94 species, the genus *Tetracha* Hope is today widely distributed in South, Central and the southern parts of North America^18–20^ (Fig. 1). Based on the taxonomic placement of the fossil specimen, Larsson^12^ concluded that *T. carolina* was widespread, occurring on both sides of the Atlantic Ocean during the Paleogene and then had perished from regions east of the ocean. The fossil was subsequently investigated by Hieke and Pietrzeniuk^21^ and more intensively by Röschmann^22^ using light microscopy. Based on the characteristic stratigraphy of the amber piece and syn-included plant remains (stellate hairs) the latter author found clear evidence that the fossil specimen is not a forgery as had been suspected, e.g., by Cockerell^23^. However, while comparing the fossil with extant specimens of *T. carolina*, Röschmann^22^ noted some morphological differences that led him to conclude that it may represent a separate species of *Tetracha*. He announced the description of the new *Tetracha* species in a manuscript cited as “Röschmann & Grimaldi 1999, in press”, which was, however, never published.

For the following two decades, the amber piece bearing this fossil cicindelid specimen disappeared without a trace and was believed to have been lost^24^. Fortunately, in 2020 it was returned to its holder, the Natural History Museum, Berlin (NMB). Thanks to the courtesy of Christian Neumann (NMB) we had the opportunity to study Horn’s Baltic amber cicindelid fossil immediately after its return to the museum.

As previously described by Röschmann^22^, the amber piece was originally cut in the shape of a flat antique vase with a size of about 33 × 14 × 5 mm (Fig. 1) and was probably used as jewellery before finding its way into the hands of a palaeontologist. Although the fossil is only enclosed laterally by a relatively thin layer of amber, diagnostic details of the beetle are scarcely visible using light microscopic techniques because large regions of the beetle’s surface are covered by a patina of flow lines, synincluded dust particles, small air bubbles, and corrosion cracks (suppl. Figures 1, 2, 3). We examined the fossil using X-ray microscopy and 3D imaging techniques^25,26^. Our re-study of this and another Eocene cicindelid, the recently described *Palaeoiresina cassolai* Wiesner, Will and Schmidt^27^, yielded completely new insights into the systematic position of Horn’s tiger beetle specimen that are of particular importance for insect paleontology and time calibration of adephagan beetle phylogenies.

## Results

**Figure 2:**
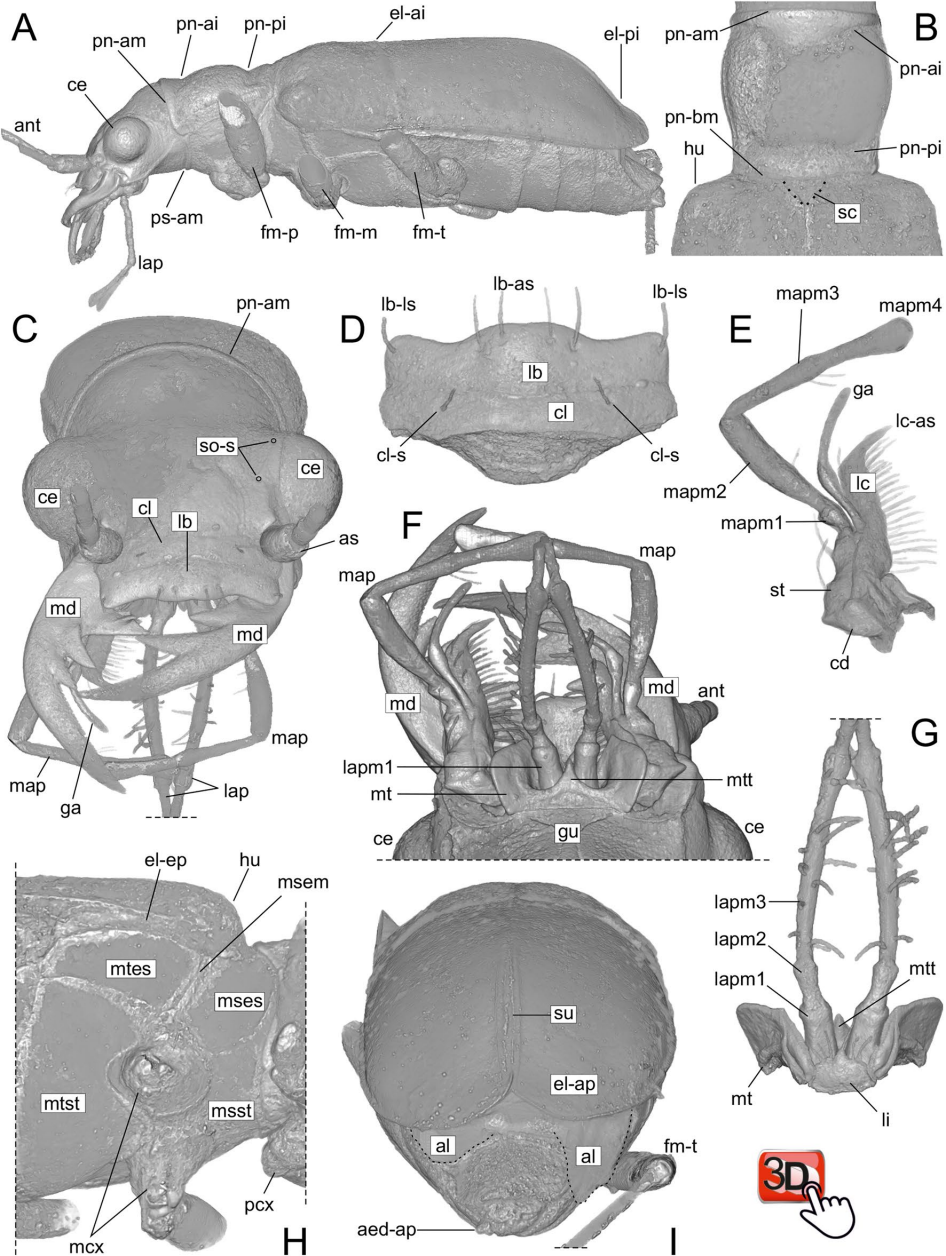
Volume rendering of Horn’s tiger beetle fossil (*Palaeoiresina cassolai* Wiesner et al., 2017) with specimen ID MB.J 1647 in the NMB. (**A**) beetle body, left lateral view. (**B**) pronotum and anterior part of elytra. (**C**) head, frontal view. (**D**) labrum and clypeus, dorsal view. (**E**) right maxilla, ventral view. (**F**) anterior part of head, ventral view. (**G**) labium, dorsal view. (**H**) meso- and metathorax, right latero-ventral view. (**I**) apical part of elytra and abdomen, caudal view. Abbreviations: aed-ap, apex of aedeagal median lobe; al, hindwings (highlighted by dotted line); as, antennal scape; ant, antenna; cd, cardo; ce, compound eye; cl, clypeus; cl-s, clypeal setae; el-ai, elytral anterior impression; el-ap, apex of elytra; el-ep, elytral epipleuron; el-pi, elytral posterior impression; fm-p, -m, -t, pro-, meso-, metafemur; ga, galea; gu, gula; hu, humerus; la, labrum; la-as, -ls, apical resp. lateral setae of labrum; lap, labial palpus; lapm1, 2, 3, labial palpomeres 1, 2, 3; lc, lacinia; lc-as, apical spur of lacinia; li, ligula; map, maxillary palpus; mapm1, 2, 3, 4, maxillary palpomeres 1, 2, 3, 4; mcx, mesocoxa; md, mandibles; msem, mesepimeron; mses, mesepisternum; msst, mesosternum; mt, mentum; mtes, metepisternum; mtst, metasternum; mtt, median tooth of mentum; pcx, procoxa; pn-am, anterior margin of pronotum; pn-bm, basal margin of pronotum; pn-ai, pronotal anterior impression; pn-pi, pronotal posterior impression; ps-am, anterior margin of prosternum; sc, scutellum (highlighted by dotted line); so-s, insertion points of the supraorbital setae (only shown on left side of head); st, stipes; su, elytral suture. The interactive PDF of the microCT reconstruction of the head of Horn’s tiger beetle fossil as an embedded 3D image is available at Supplementary figure 2.

**Figure 3:**
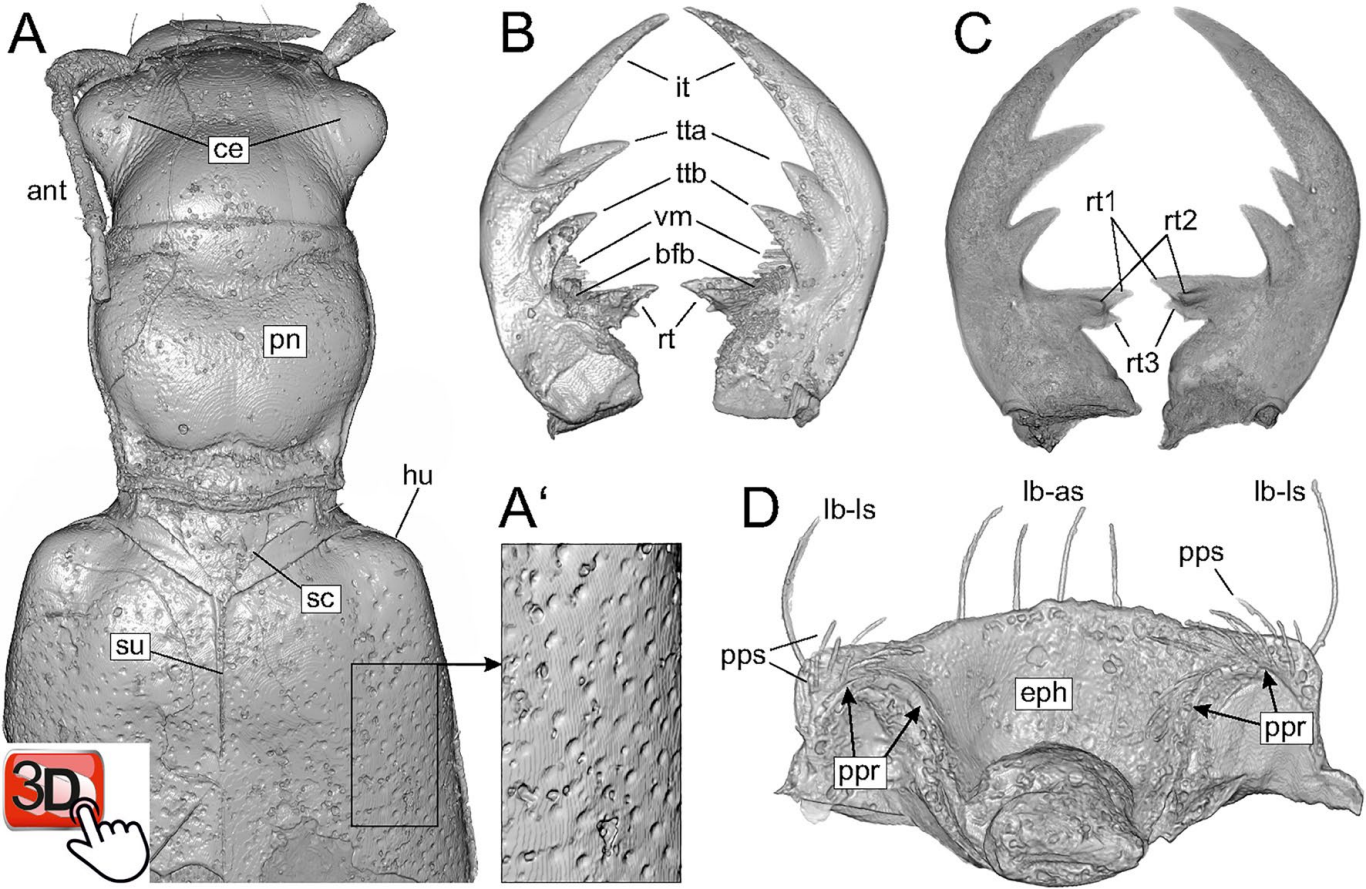
Volume rendering of the holotype specimen of *Palaeoiresina cassolai* Wiesner et al., 2017 (**A**, **B**, **D**) and Horn’s tiger beetle fossil (**C**). (**A**) anterior part of beetle body, dorsal view, with (**A**’), enlarged section of part of the elytra, showing puncture. (**B**, **C**) mandibles, dorsal view. (**D**) ventral surface of labrum. Abbreviations: ant, antenna; bfb, basal face brush of mandible; ce, compound eye; eph, epipharynx, dorsal surface; hu, humerus; it, incisor tooth; la-as, -ls, apical resp. lateral setae of labrum; pn, pronotum; ppr, parapedial ridge; pps, parapedial setae; rt, retinacular tooth; rt1, 2, 3, retinacular tooth, cusp 1, 2, 3; sc, scutellum (highlighted by dotted line); su, elytral suture; tta, ttb, anterior resp. basal terebral tooth; vm, ventral microtrichia of mandible. The interactive PDF of the microCT reconstruction of the forebody of the holotype specimen of P. cassolai as an embedded 3D image is available at Supplementary figure 3.

The investigated fossil specimens are documented in Figs. 2, 3 and suppl. Figures 1–3. Measurements are recorded in suppl. Table 2. Both the fossil specimens, Horn’s tiger beetle and the holotype of *Palaeoiresina cassolai*, share the characteristics of Cicindelidae as defined by Arndt et al.^28^ and Ball et al.^29^ as well as the following diagnostic character states:

- **Head:** Head width including eyes distinctly broader than pronotum. Dorsal and lateral surfaces without additional setae or hairs beside primary setation. Supraorbital area and temples with several moderately deep engraved longitudinal furrows. Compound eyes large, moderately strongly protruded laterally. Two supraorbital setae present each side in normal position for tiger beetles^30^. Clypeus bisetose. Labrum transverse, dorsally six-setose, with lateroapical seta present on each side. Apical margin of labrum slightly convexly protruded in middle, without any tooth-like structures. Epipharynx on ventral side of labrum with the row of anterior parapedial setae extended laterally, parallel to the lateral extensions of parapedial ridge^29^. Antennal scape large, with a single seta near apical margin. Mandibles robust, with external surface glabrous (without scrobal setae), with long incisor tooth (incisor of right mandible slightly longer than left one), with two terebral teeth, without diastema between retinaculum and basal terebral tooth^29^, with basal terebral tooth monocuspidate, with retinaculum quadricuspidate, and without supplementary retinacular tooth; the anterior terebral tooth is shorter than the posterior one in the right mandible and larger in the left mandible. Maxilla with apical spur of lacinia large; maxillary palps with palpomere 2 longest, and palpomere 3 as long as or very slightly longer than 4. Apical palpomeres of maxillae and labium glabrous. Labium with palpiger covered by mentum if viewed ventrally, with base of labial palpomere 1 more posteriorly situated than mentum notch^31,32^. Labial palps distinctly longer than maxillary palps, with palpomere 3 longest, as long as or only slightly longer than 4; palpomere 1 longer than second. Mentum deeply notched laterad of mentum tooth; latter moderately slender, acute, shorter than lateral lobes of mentum.
- **Pronotum:** Pronotum about as long as broad, slightly narrower than head, with lateral margin distinct, complete. Pronotal apical margin projected anteriorly, beyond the prosternal apical margin. Pronotal basal margin almost as broad as apical margin, slightly sinusoidal, with posterior margin of laterobasal angles situated at level of middle portion of pronotal basal margin. Proepisternum with transversal furrow rather shallow. Prothoracic surface smooth and glabrous.
- **Pterothorax:** Scutellum large, not covered by pronotal base. Elytra very slender ovate with humerus well-developed, in lateral view with a very shallow transverse impression just anterior to the midpoint of the length, and a second transverse impression before apex; apices broadly rounded. Elytral surface with sparse setation in anterior third (somewhat denser in the humeral area) and extensive punctures; punctures are moderately large in anterior quarter and gradually smaller towards elytral apex. Hind wings fully developed. Mesepisternum broad. Metepisternum longer than wide but not markedly elongated, without impressions. Pterothoracic plates glabrous.
- **Abdomen:** Surface of sternites smooth, without pilosity or rugosity; sternites 3–5 each with two setae in middle near apical margin (setae could not be imaged by CT settings used but are visible using light microscopy).

### Morphological differences between the fossil specimens

Specimen MB.J 1647 differs from the *P. cassolai* holotype by larger body size (standardized body length 11.7 compared to 9.6 mm) and more markedly protruded compound eyes (head width including eyes/ head width between eyes = 1.71 compared to 1.57).

## Discussion

### Taxonomic position of Horn’s tiger beetle fossil in the genus *Palaeoiresina*

The extensive morphological similarity of the tiger beetle fossil MB.J 1647 with the *P. cassolai* holotype, which includes all diagnostic data for unequivocal taxonomic assignment within Cicindelidae, is evidence that Horn’s tiger beetle fossil should be placed in the genus *Palaeoiresina*. The two evident morphological differences between the fossil specimens, body size and compound eye convexity, are not considered sufficient to distinguish fossil MB.J 1647 and *Palaeoiresina cassolai* as representing separate species. It is likely that these differences fall in the range of intraspecific variability. E.g., the body length of specimen MB.J 1647 is 1.22 times of that of the *P. cassolai* holotype. In some recent species of the genus *Oxycheila* Dejean, greater differences in the body size were measured, e.g., the largest individuals up to 1.31 times the length of the smallest in specimens of *O. tristis* (Fabricius, 1775)^33^. Moreover, considering the longevity of the Eocene species, which may have amounted to several million years, the divergent development of body size or eye convexity seems very plausible. Therefore, based on the current data set, we propose the following:

*Palaeoiresina cassolai* Wiesner, Will & Schmidt, 2017.

 = *Tetracha carolina* cited by Horn (1906)^17^, non *T. carolina* (Linnaeus, 1763)^34^.

 = *Tetracha “*new species” cited by Röschmann^22^.

### Evidence for the recognition of *Palaeoiresina* as separate from *Tetracha* and Megacephalini cicindelids

Based on the results of molecular phylogenetic studies in Cicindelidae, six monophyletic groups are recognized and tribal level taxa were proposed for each: Cicindelini, Ctenostomatini, Collyridini, Manticorini, Megacephalini, and Oxycheilini^35^. Adults of the tribe Megacephalini in the sense of Arndt and Putchkov^36^ and Duran and Gough^35^, which includes the genus *Tetracha*, are characterized by two derived characters: (1) absence of the latero-marginal seta both sides of labrum and (2) presence of a supplementary retinacular tooth^19,29^. Regarding both these diagnostic features, in *Palaeoiresina* we found the plesiomorphic pattern (Figs. 2, 3). Therefore, *Palaeoiresina* lacks the synapomorphies of the Megacephalini crown group. Duran and Gough^35^ and Zhao et al.^37^ noted three additional character states to define Megacephalini, (1) the transverse labrum with (2) toothless apical margin and (3) the anteriorly projected pronotal margin, all are likewise present in *Palaeoiresina*. However, these states are plesiomorphic within Geadephaga and cicindelids^29,38^, and so provide no information about relationships within the family.

### Placement of *Palaeoiresina* relative to the Megacephalini + Collyridini + Ctenostomatini + Cicindelini + Oxycheilini clade

**Figure 4:**
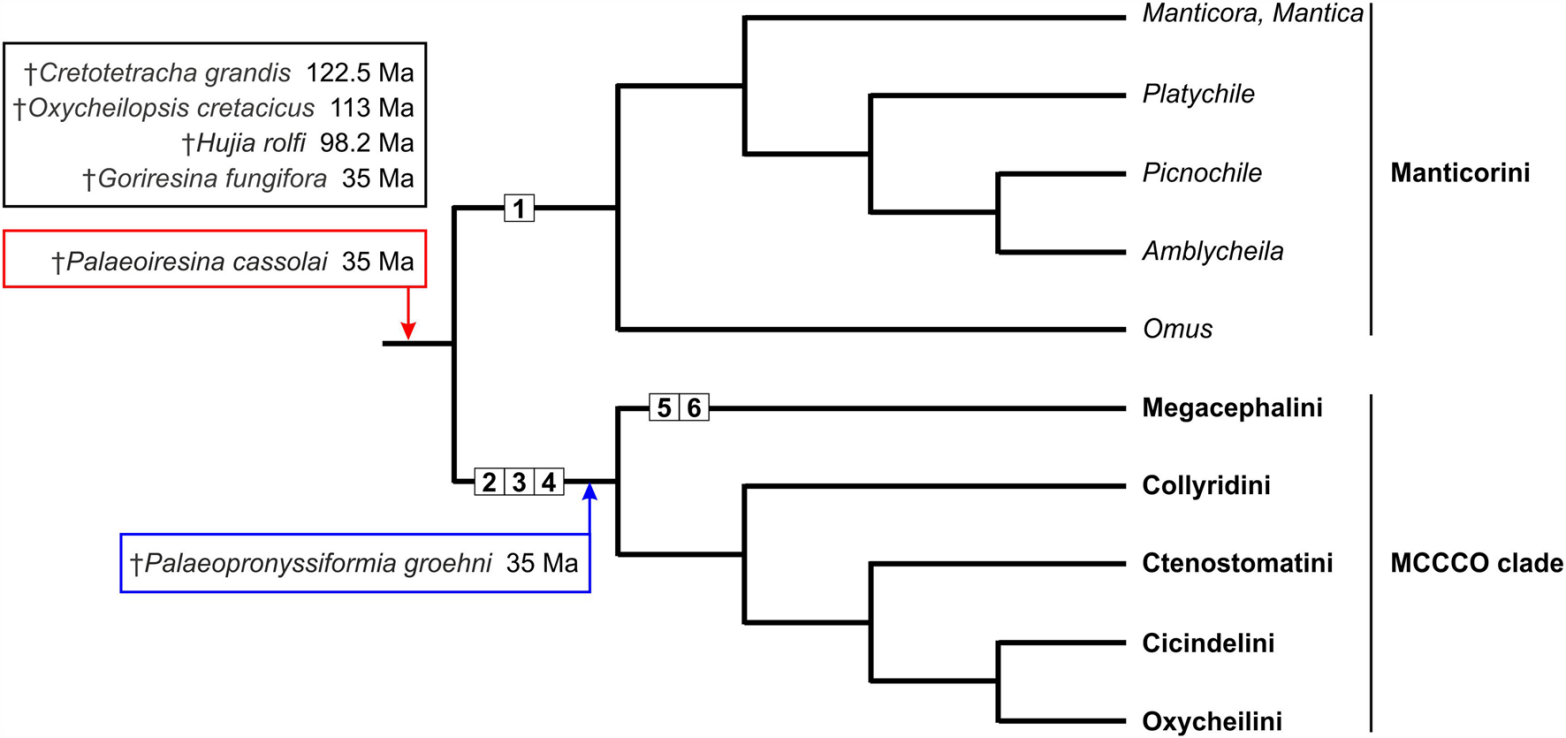
Molecular genetic based phylogeny of Cicindelidae as presented by Duran and Gough^35^ with *Mantica* added, and with reference to the morphological character states of the basal clades and the fossil representatives of the family, together with their minimum ages (see text, for details). Numbers in white boxes refer to synapomorphies of the clades: **1** basal terebral tooth tricuspidate. **2** mandibular diastema developed. **3** anterior epipharyngial parapedial setae terminally detached from the parapedial ridge. **4** labial palpiger not covered by the mentum. **5** absence of the latero-marginal seta both sides of labrum. **6** presence of a supplementary retinacular tooth. Of the six currently-known fossil species of cicindelids, one is identified as being stem group member of the family (red box), one of the MCCCO clade (blue box), while the systematic positions of four taxa are unknown (black box).

Molecular studies have consistently supported the results of a previous phylogenetic analyses based on larval morphology, showing a monophyletic clade that includes all Cicindelidae except Manticorini. This clade, hereafter referred to as the MCCCO clade, includes Megacephalini as sister to (Ctenostomatini, (Collyridini, (Cicindelini , Oxycheilini)))^36,39–42^; Fig. 4). Each of the MCCCO clade tribes is characterized by derived, adult character states that are found to be plesiomorphic in *Palaeoiresina*^31,32,35^. *Palaeoiresina* shares with most species of the MCCCO clade the large and bulging eyes (except for the presumed derived smaller eyes of species known to be nocturnal^35^). However, this character state is possibly plesiomorphic for tiger beetles as large eyes are also found in some Manticorini. Additionally, more or less well-developed eyes are known for taxa from across Geadephaga, a pattern of homoplasy that suggests limited phylogenetic weight should be put on this character. Ball et al.^29^ described two derived character states for members of the MCCCO clade: (1) mandibles with a more or less extensive occlusal diastema between anterior margin of retinaculum and basal terebral tooth (the supplementary retinacular tooth of Megacephalini and *Therates* Latreille is situated in this diastema); (2) the terminal part of the row of anterior parapedial setae of the epipharynx is detached from the parapedial ridge and extended anteriorly. In *Palaeoiresina*, we found the respective plesiomorphic pattern with absence of a mandibular occlusal diastema, and with the row of anterior parapedial setae extended laterally, parallel to the lateral extensions of the parapedial ridge^29^ (Fig. 3). Here we point to a third, probable synapomorphy of the MCCCO clade that Horn^31^ originally used to differentiate some genera of Manticorini (his subtribe Omina) from species of Megacephalini and Oxycheilini: in ventral view, the labial palpiger is covered by the mentum, because the base of palpomere 1 is more posteriorly situated than the posterior arc of the mentum notch. We found this character state in *Palaeoiresina* (Fig. 2) and in species of the genera *Manticora* Fabricius and *Platychile* MacLeay that, together with Horn’s Omina, form the Manticorini^35^ (suppl. Figure 4). In MCCCO clade species, the palpiger is situated more anterad with respect to the mentum notch and is thus clearly visible when viewed ventrally (suppl. Figure 4). In this position, the first labial palpomere has a greater degree of freedom and can be laid backwards by more than 90° relative to mentum. In the other two families of Geadephaga, the labial palpiger and base of palpomere 1 are uncovered (Trachypachidae, several groups of Carabidae, e.g., Nebriini, Omophroninae, Trechinae, Harpalinae) or covered by the mentum (other Carabidae, e.g., Carabinae, Elaphrini, Loricerinae, Notiophilinae, Pelophilini, Brachinini), with intermediate states, e.g., in Scaritinae (suppl. Figure 4). However, independent of the position of the palpiger, the range of motion of the first labial palpomere in trachypachids and most carabid beetles is more limited, and most of the dorso-ventral movement of the palpus in these groups is realized by the second or third palpomere. Based on these observations we hypothesize that a fully free labial palpomere 1 (labial palpiger not covered by the mentum) with markedly high degree of mobility is a third derived character state of the MCCCO clade. Because *Palaeoiresina* does not share any of the three MCCCO apomorphies, it is very unlikely that this fossil species is a crown group member of this clade of Cicindelidae.

### Evidence against Palaeoiresina being a member of Manticorini

The hypothesis of a monophyletic tribe Manticorini that includes *Manticora*, *Omus* Eschscholtz, *Amblycheila* Say, *Picnochile* Motschulsky and *Platychile* and is the sister group to all remaining tribes of extant Cicindelidae (the MCCCO clade, see above) is mainly supported by molecular data of the 18S rRNA locus^35,39,41^. The monotypic genus *Mantica* Kolbe was not included in any of the molecular studies. However, there has never been any question as to the close relationships of the South African *Mantica horni* Kolbe and *Manticora*^35^. Duran and Gough pointed out that no morphological synapomorphic feature is known for the Manticorini as circumscribed using molecular data. Morphological studies of larvae^36^ and imagines^29^ resulted in a different phylogeny, with a paraphyletic Manticorini and basal grade of *Omus*, *Amblycheila* and *Picnochile* (*Mantica* and *Platychile* were not included). Ball et al.^29^ proposed the tribe Amblychilini for the New World genera *Omus*, *Amblycheila* and *Picnochile* based on presence of two additional cusps on the basal terebral tooth. The molecular genetic Manticorini concept^35^ places *Omus* sister to the other lineages of the tribe, and *Amblycheila* and *Picnochile* in a more derived position than *Manticora* and *Platychile*. The phylogeny based on molecular data implies that that the character state ‘tricuspidate basal terebral tooth’ was most likely lost twice in Manticorini evolution, separately in the *Manticora* and *Platychile* lineages. This reconstruction of the character state transition as two losses is consistent with the following observations: In *Platychile*, a marked dorsal ridge is developed near base of the basal terebral tooth that can be interpreted as representing a derived state of the dorsal-most of the two secondary cusps (suppl. Figure 4). In *Manticora*, the mandibular morphology is highly derived, with species-specific, sexual dimorphism. However, in the closely related but little studied *Mantica*, we found a tricuspidate basal terebral tooth similar to what is found in *Omus* and *Amblycheila*^29^ (suppl. Figure 4). Consequently, the evolution of a tricuspidate, basal terebral tooth in the Manticorini stem group is a very likely scenario, and this character state is considered a synapomorphy of the tribe. Given that *Palaeoiresina* has a monocuspidate, basal, terebral tooth, the plesiomorphic state for the family (Figs. 2, 3), there is no evidence that it is member of crown Manticorini.

### Taxonomic position of additional cicindelid fossils

The review of synapomorphies of cicindelid tribes and their inclusive clades above makes it possible to critically discuss the previous opinions and evidence for the systematic position of the five additional, described cicindelid fossils from pre-Neogene deposits. All these fossils share two synapomorphic character states of the family^29^: (i) hypertrophied incisor tooth and terebral teeth of the mandibles and, (ii) by the development of a second terebral tooth^27,37,43–45^.

1. *Cretotetracha grandis* Zhao et al. was described from sedimentary rocks of the Yixian Formation, Inner Mongolia, China (124–122.5 Ma) and attributed to Megacephalini due to its similarity with recent species of that tribe. Characteristics considered evidence of this placement include body size and proportions, transverse labrum with toothless apical margin, large eyes, and apical margin of pronotum noticeably more extended anteriorly than apical margin of prosternum^37^. As discussed above, none of these character states provides clear evidence for placement in Megacephalini because they are either plesiomorphic within Cicindelidae or are found to occur independently in several cicindelid clades. Instead, the images of the *C. grandis* mandibles presented by Zhao et al.^37^ (Fig. 3) clearly show, that neither a diastema nor a supplementary retinacular tooth is developed in the fossil specimen. Consequently, *C. grandis* cannot be considered a member of Megacephalini or of the MCCCO clade. As it lacks synapomorphic character states of these recent Cicindelidae clades, *C. grandis* must be considered a representative of an extinct lineage of Cicindelidae with unknown systematic position outside the MCCCO clade (Fig. 4).
2. *Oxycheilopsis cretaicus* Cassola & Werner from calcerous sediments of the Crato Formation, Ceara, Brazil (119–113 Ma) is a beetle with some unusual characters (for Cicindelidae) of the head and tarsal morphology. It was described as a member of Oxycheilini due to similarities in general facies, body proportions and pronotal shape^45^. However, diagnostic characters for Oxycheilini are not visible in this compression fossil, and none of the character states that define any of the present cicindelid lineages were found. Therefore, *O. cretaicus* is a fossil species with unknown systematic position within Cicindelidae (Fig. 4), i.e., Cicindelidae incertae sedis.
3. Recently, *Hujia rolfi* Song et al. was described from Burmese amber, Kachin State, Myanmar (98.8 + /- 0.6 Ma)^44^. The authors place this fossil in the tribe Manticorini based on its lack of brightly coloured areas of body, small and flat eyes, and similar length of incisor and terebral teeth of mandible compared to that of the recent species *Amblycheila baroni* (Rivers) from southern North America. This placement is questionable for several reasons. First, presence of two additional cusps on the basal terebral tooth is the only known synapomorphic feature of Manticorini (see discussion above) but this was not observed in the fossil. Second, dark body colour and small compound eyes are character states homoplastically found among species of Cicindelidae that can be explained by convergence of nocturnally activity beetles. Therefore, the phylogenetic evidence provided of these states alone must be considered low. Third, evidence provided by the lengths of mandibular incisor and terebral teeth is suspect as this varies markedly within Manticorini^29^ making discrete states and homology difficult to justify. Moreover, the authors opine that the mandibular morphology of *H. rolfi* is similar to *Amblycheila* but the evident diagnostic character states differ for these taxa: In *Amblycheila*, the mandibular scrobe is multisetose but asetose in *H. rolfi*. In *Amblycheila* and most Manticorini lineages, the pronotal apical margin is projected anteriorly, beyond the prosternal apical margin. In *H. rolfi*, pronotal and prosternal apical margins are on the same level as found in Cicindelini, Collyridini, and Ctenostomatini. The only Manticorini lineage with similar pronotal morphology is *Manticora,* which has, however, a multisetose mandibular scrobe. Consequently, the morphological evidence does not place *H. rolfi* in Manticorini or in any of the recent Cicindelidae tribes. At our current state of knowledge, *H. rolfi* is yet another fossil species with an unknown systematic position within Cicindelidae (Fig. 4), i.e., Cicindelidae incertae sedis.
4. The fossil *Palaeopronyssiformia groehni* Wiesner et al. was described from Eocene Baltic amber and due to its poor preservation condition, tentatively attributed to the subtribe Iresina of the tribe Cicindelini^27^. Although monophyly of Cicindelini is strongly supported by multiple molecular phylogenetic analyses^39–42^, morphological synapomorphies of this highly diverse group are unknown. Morphological synapomorphic character states are also unknown for the subtribe Iresina. This situation prevents unequivocal assignment of fossils and recent taxa that have not been sequenced to Cicindelini. Given the mandibles of *P. groehni* show a distinct diastema posterior of the basal terebral tooth^27^, this fossil may be considered member of the stem lineage of the MCCCO clade (Fig. 4).
5. *Goriresina fungifora* Matalin & Perkovsky & Vasilenko was described from Eocene Rovno amber and likewise attributed to Iresina of the Cicindelini tribe^43^. The fossil was only examined using light microscopy. Mandibles and basal parts of the labium are not visible using this technique. Therefore, diagnostic character states that allow for unequivocal assignment of the fossil to any of the Cicindelidae tribes are not available. Its systematic position remains unknown. Micro-Xray scanning, as used here, will be a suitable tool to investigate this fossil and hopefully settle the systematic position of *G. fungifora*.

## Conclusions

It was probably the similarity in the general habitus of the Baltic amber cicindelid with the extant species of *Tetracha*, that led Walther Horn^17^ and subsequent authors to believe that the fossil was conspecific with *T. carolina* or, alternatively, a separate species within this Nearctic genus^12,16,22^. However, the detailed micro-CT based reconstruction of the external morphology of the fossil shows evidence that places Horn’s tiger beetle specimen in the genus *Palaeoiresina* and conspecific with the fossil species *P. cassolai*, a specimen of which was found in the same amber deposit. In addition, reconstructed character states of the labrum, mandibles, epipharynx, and labium of both fossil specimens show that *Palaeoiresina* is neither a member of the tribe Megacephalini, in which the genus *Tetracha* is placed, nor a member of the megadiverse MCCCO clade, which includes 99% of the extant tiger beetle species^46^. Given the absence of any of the apomorphic features found in Manticorini mandibles, we conclude, that *Palaeoiresina* is not a member of the crown group of this tribe, which includes all the remaining lineages of recent cicindelids. Based on the morphological data, *Palaeoiresina* cannot be placed in the crown groups Manticorini or the MCCCO clade. Most likely, *Palaeoiresina* represents an extinct stem group that is the lineage including the common ancestor and all modern Cicindelidae (Fig. 4). However, it cannot be ruled out that the fossil is a member of a stem group of one of either of the two tiger beetle clades, Manticorini or the MCCCO clade, that underwent separate evolution before the crown group autapomorphies evolved.

The implications of our study go well beyond simply correcting the taxonomic placement of any particular fossil tiger beetle species. Our results are highly relevant for using this Baltic amber fossil to calibrate divergence dating of beetle phylogenies. Using fossils that are phylogenetically incorrectly placed or have their age misspecified introduces significant error into the analysis^47^. Apomorphy-based placement of fossils for dating is crucial as the possible placements significantly affect the minimum age of the clades in question and the error around such estimates. Our investigation of the morphology of the relevant taxa and available fossils provides evidence that rejects the idea of a recent species of Cicindelidae persisting since the Eocene period. Horn’s “*Tetracha carolina*” fossil, which we recognized as representing a *Palaeoiresina* species, could only be used to date the stem lineage of Cicindelidae. However, the minimum evolutionary age of cicindelid beetles is already known to be much older than Baltic amber, dating back to the Early Cretaceous (122.5 Ma) based on the cicindelid fossil *Cretotetracha grandis*^37^ (Fig. 4). The morphological data presented here and in previous publications describing cicindelid fossils^27,37,43–45^ also shows that the assignment of these tiger beetle fossil species to extant lineages was not based on synapomorphies but rather on general similarity of form or phenetic character similarities shared with recent species. These previous hypotheses regarding the systematic position of these fossils are not supported. There is currently no fossil evidence for the presence of Manticorni or Megacephalini cicindelids in the Cretaceous period, nor for members of the Iresina subtribe of Cicindelini in the Eocene.

Finally, our results for *Palaeoiresina cassolai* and an absence of convincing evidence for other putative old, extant insects, is another important indication that the maximum age of extant insect species most likely does not go back to the Paleogene. The evidence-based placement of this Baltic amber tiger beetle fossil corrects what was proposed by Horn^17^ and subsequent authors, removing the last suspicion of an Eocene beetle fossil belonging to a recent species. Other Coleoptera fossils that have been attributed to species living today are from the Neogene period, with the oldest are noted from the Dominican and Mexican amber (about 20–15 Ma;^4,48^ or 15.75–12.58 Ma^49^). Consequently, and according to the age estimation for insect species proposed by Grimaldi and Engel^7^, all the additional fossil findings from the Paleogene period that have been attributed to recent species, need to be critically reviewed.

## Material and methods

### Investigated fossil material

1. Horn’s tiger beetle fossil in Baltic amber, with specimen label data “MB.J 1647 / Paläontologisches Museum Berlin / Tetracha carolina L. / (Coleoptera: Cicindelidae) / vid. HIEKE, 1983 / Baltischer Bernstein / Slg. Berendt”, deposited in NMB. Size of the piece approx. 33 × 14 × 5 mm (maximum values, irregularly cut; Fig. 1). Micro-CT scanning was performed with a voxel size of 7.4 µm for the whole beetle body, and a detailed scan of the head was performed with a voxel size of 4.4 µm. The fossil is rather poorly preserved, with the surface of the beetle inclusion covered by corrosion cracks, flow lines, synincluded dust particles, small air bubbles and a patina that appears strongly refractive in CT. Most parts of the antennae and legs are lost due to ruthless cutting of the original amber piece (suppl. Figures 1–3). Micro-setae of mandibles and epipharynx as well as internal structures of the beetle body including genitalia are not preserved; only the tip of the aedeagal median lobe which protrudes slightly from the apical segment of the abdomen, is visible using CT (Fig. 2). However, head with mouth parts provide sufficient contrast in CT (Fig. 2); the apical portion of the left lacinia is not preserved (lacinia may have already been damaged while the beetle was alive).
2. *Palaeoiresina cassolai* Wiesner, Will & Schmidt, 2017, fossil in Baltic amber; holotype, male, with specimen number UCMP404030, locality number UCMP IP15208, in the University of California, Museum of Palaeontology, Berkeley, CA; restudy of the specimen using the micro-CT data obtained by Wiesner et al.^27^. These scans were performed with a voxel size of 4.3 µm for the head and 4.6 µm for the pterothorax. The anterior part of the fossil is moderately well preserved and provides good contrast in CT (Fig. 3). Parts of the abdomen are lost due to amber corrosion; for details see fossil documentation in Wiesner et al.^27^.

### Investigation techniques

The MicroCT raw data (tiff stacks) used in the current study are available in the Dryad data repository, 10.6078/D1VH9M. The fossils were studied and imaged via light microscopy and micro-computed tomography (micro-CT) using the Leica M205-C stereomicroscope with Leica DFC450 digital camera and the Xradia 410 Versa-X-ray microscope (Zeiss, Pleasanton, CA, USA). These methods were described in detail in our previous papers^25,26^. For details of the light microscopic results of *P. cassolai* see Wiesner et al.^27^. Micro-CT scan settings used for 3D imaging of the specimens are shown in suppl. Table 1. Volume rendering of image stacks was performed by using Amira 6.6 software (FEI Visualization Science Group, Burlington, USA) using the “Volren”, “Volume Rendering”, and “Segmentation” functions. CT-based morphological investigations were carried out using the “Volren”, and “Volume Rendering” functions of Amira. These and the “Segmentation” functions of Amira were used for processing the figures of the morphological details as shown in Fig. 2 (A–I) and Fig. 3 (A–D).

### Generation of 3D pdfs

Surface objects were performed using the “Generate surface” function in Amira 6.6 and exported to Meshlab v2020.02 open-source mesh processing tool^50^. We used Meshlab for (i) cleaning the 3D-modells via removal of duplicate faces/vertices and unreferenced vertices, repairing of non-manifold edges by removing faces and zero area faces, (ii) smoothing with Taubin and Laplacian smooth and (iii) simplification via Quadric edge collapse decimation in several steps to prevent loss of fine structures of the 3D-models. Surface objects were then reorganized and finished via Deepexploration v5.5 software (former Right Hemisphere). The final pdf embedding was done with Adobe Acrobat 9 Pro Extended software (Adobe Systems Incorporated).

### Measurements

The measurement software of Amira was used and applied to the X-ray scanning results of the fossil. For details of the measurement methods for the different body parts see suppl. Table 2.

### Supplementary Information

Supplementary Information.

Supplementary Figure 1.

Supplementary Figure 2.

Supplementary Figure 3.

## Data Availability

All data needed to evaluate the conclusions in the paper are present in the paper and/or the Supplementary Materials. The image stacks of the micro-CT scans have been deposited in Dryad and accessible via this link: 10.6078/D1VH9M.
