# Supplementary material for: MicroCT data provide evidence correcting the previous misidentification of an Eocene amber beetle (Coleoptera, Cicindelidae) as an extant species
Source: Sci Rep. 2023 Sep 7;13:14743. doi: 10.1038/s41598-023-39158-7 (PMC10484930; doi:10.1038/s41598-023-39158-7)
Supplement: Supplementary file 1 — Supplementary Information. [file 41598_2023_39158_MOESM1_ESM.pdf]

MicroCT data turn taxonomy upside down:
The mystery of Walter Horn's Baltic amber tiger beetle solved (Coleoptera: Cicindelidae)

Supplementary information

Table S1. MicroCT scan settings applied to the investigated *Palaeoiresina cassolai* fossil specimens from Baltic amber.

**scan settings**	**MB.J 1647** total beetle body	head	**UCMP404030 (holotype)** head	pterothorax
voltage [V]	40	50	40	40
power [W]	8	8	8	8
object lens	0.4	4	4	4
lens filter	LE1	LE1	-	-
cam binning	1	2	2	2
distance to source [mm]	40	70	120	55
distance to detector [mm]	140	35	120	69
vertical stitch	-	-	2	-
voxel size [µm]	7.4	4.4	4.3	4.6
exposure time [sec]	25	20	20	40
number of images/ segment	981 (208°)	1001 (186°)	2401 (360°)	2401 (360°)

Table S2. *Palaeoiresina cassolai* fossil specimens from Baltic amber: details of measurement methods and measurement results.

character	measurement method	value [mm]	
		MB.J 1647	UCMP404030 (holotype)
standardized body length	equals the sum of the lengths of the head, pronotum, and the longer elytron	11.7	9.6*
length of the head	from anterior margin of clypeus to a point on the midline at level of the posterior margin of the compound eye	1.53	1.37
width of head including compound eyes	across the widest portion including compound eyes	2.80	2.33
width of head between compound eyes	across the shortest distance between compound eyes	1.64	1.48
length of the compound eye: left/right	at their widest points (lateral aspect)	1.05/1.01	8.45/8.64
width of labrum	at the widest portion	1.58	1.36
length of the antennomer 1: left/right	between most distant points	0.84/0.87	0.86/n.a.
length of the antennomer 2: left/right	between most distant points	0.33/0.35	0.30/n.a.
length of mandibular incisor tooth (left/right)	from internal base to apex (Ball et al. 2011)	0.94/1.13	0.96/1.03
length of the maxillary palpomere 2 (left/right)	between most distant points	0.95/0.90	0.81/0.81
length of the maxillary palpomere 3 (left/right)	between most distant points	0.79/0.77	0.63/0.65
length of the maxillary palpomere 4 (left/right)	between most distant points	0.73/0.75	0.63/0.63
length of the labial palpomere 3 (left/right)	between most distant points	1.27/1.25	1.09/1.09
length of the labial palpomere 4 (left/right)	between most distant points	1.24/1.22	1.16/1.15
length of pronotum	from apical to basal margin along midline	2.35	2.17
width of pronotum	at the widest portion (dorsal aspect)	2.36	2.15
pronotal apical width	between the tips of the front angles	2.07	1.86
pronotal basal width	maximum width at level of hind angles	2.00	1.70
length of elytron (left/right)	from the apex of scutellum to the apex of the elytron	7.86/7.81	6.0*
width of elytron (left/right)	at their widest points (dorsal aspect)	2.68/2.70	1.8*
length of the metepisternum (left/right)	along lateral margin	n.a./1.55	1.39/1.36
width of the metepisternum (left/right)	along anterior margin	n.a./0.95	088/0.90
length of metafemur (left/right)	between most distant points	n.a./5.02	n.a./n.a.

*Because apical portion of elytra is not preserved, the values were estimated based on the light microscopic photographs and the habitus reconstruction presented by Wiesner et al. (2017).

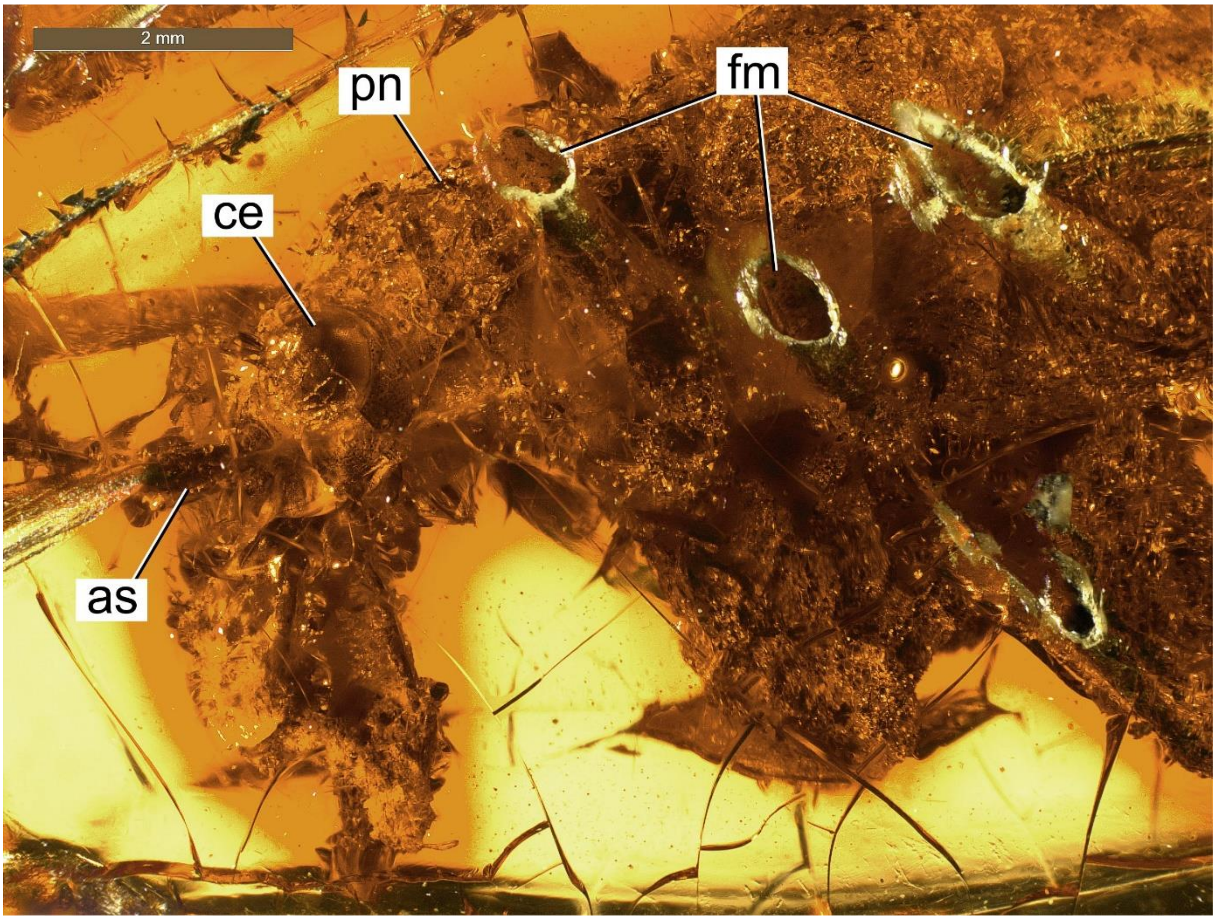

Figure S1. Light microscopic documentation of Horn's tiger beetle fossil (*Palaeoiresina cassolai* Wiesner et al., 2017) with specimen ID MB.J 1647 in the Museum of Natural History, Berlin (NMB): anterior part of the beetle body, left lateral view. Abbreviations: as: antennal scape; ce: compound eye; fm: femora (the black and white bars point to the openings of the left pro-, meso- and metafemora to the amber surface that were resulted from the amber cutting); pn: pronotum.

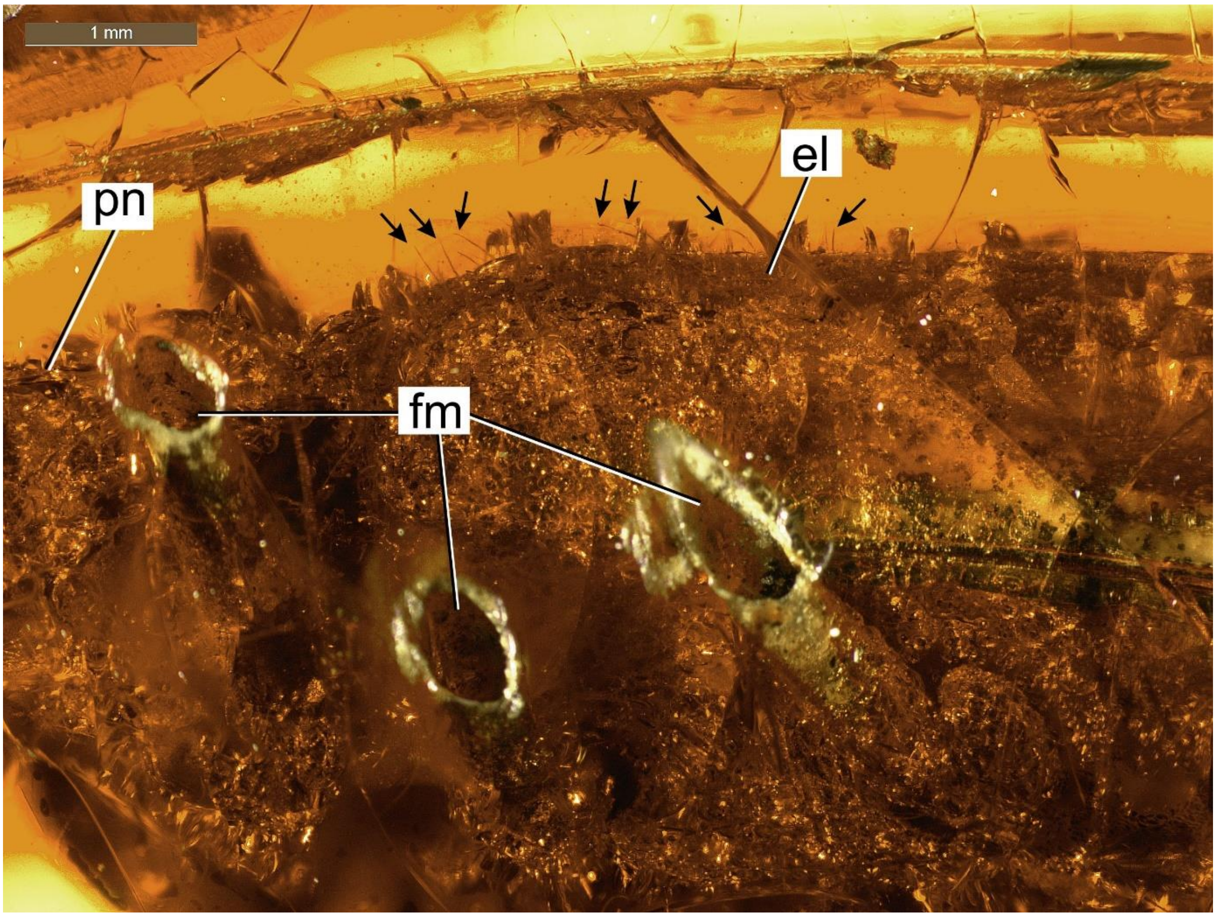

Figure S2. Light microscopic documentation of Horn's tiger beetle fossil (*Palaeoiresina cassolai* Wiesner et al., 2017) with specimen ID MB.J 1647 in the NMB: pterothorax, left lateral view. Abbreviations: el: elytron; fm: femora (the black and white bars point to the openings of the left pro-, meso- and metafemora to the amber surface); pn: pronotum. The small black arrows point to the hair-like setae which sparsely cover the anterior third of elytra.

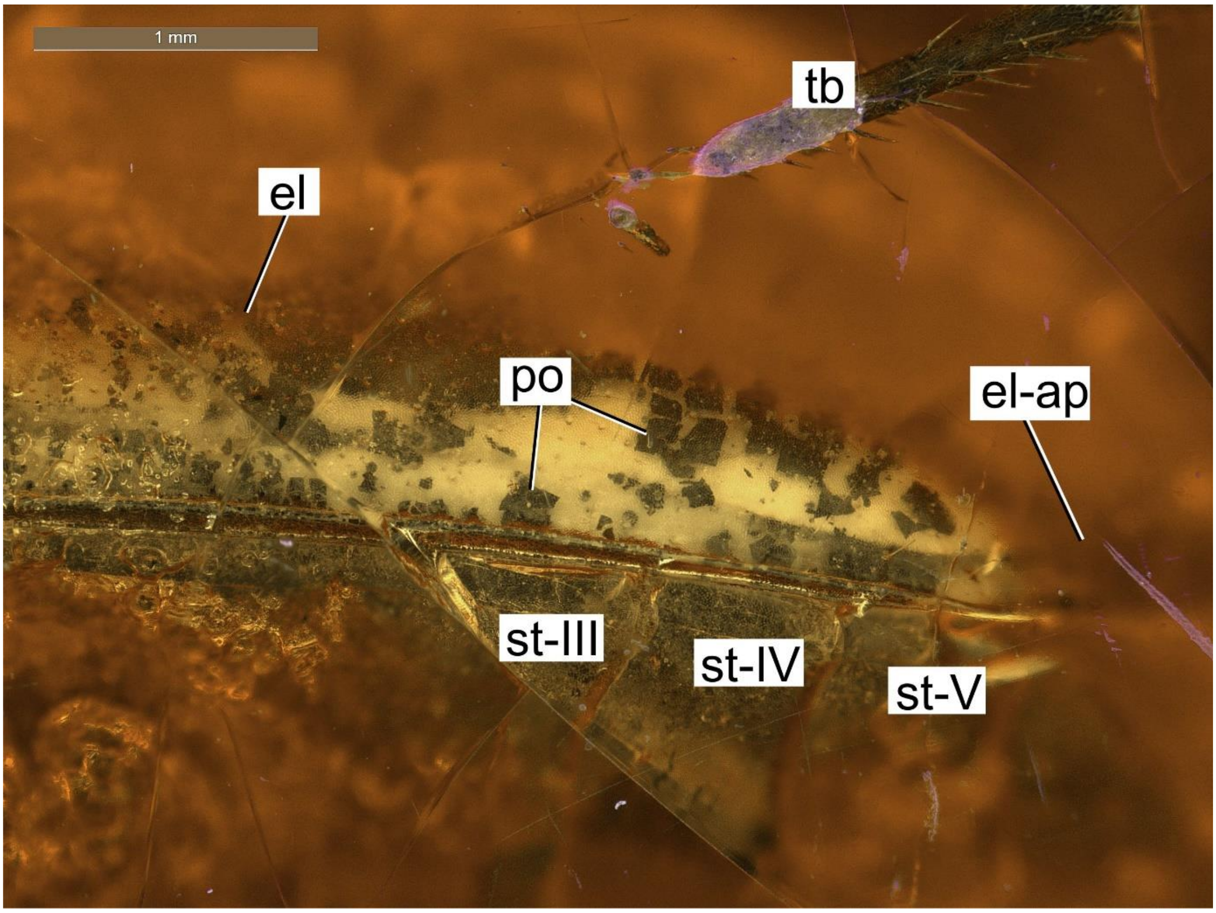

Figure S3. Light microscopic documentation of Horn’s tiger beetle fossil (*Palaeoiresina cassolai* Wiesner et al., 2017) with specimen ID MB.J 1647 in the NMB: apical portion of left elytron, left lateral view. Abbreviations: el: elytron; el-ap: elytral apex; po: fragments of the positive of the fossilized elytron, showing that the color of elytra was probably dark greenish; st-III, IV, V: abdominal sternites III-V; tb: fragment of the metatibia.

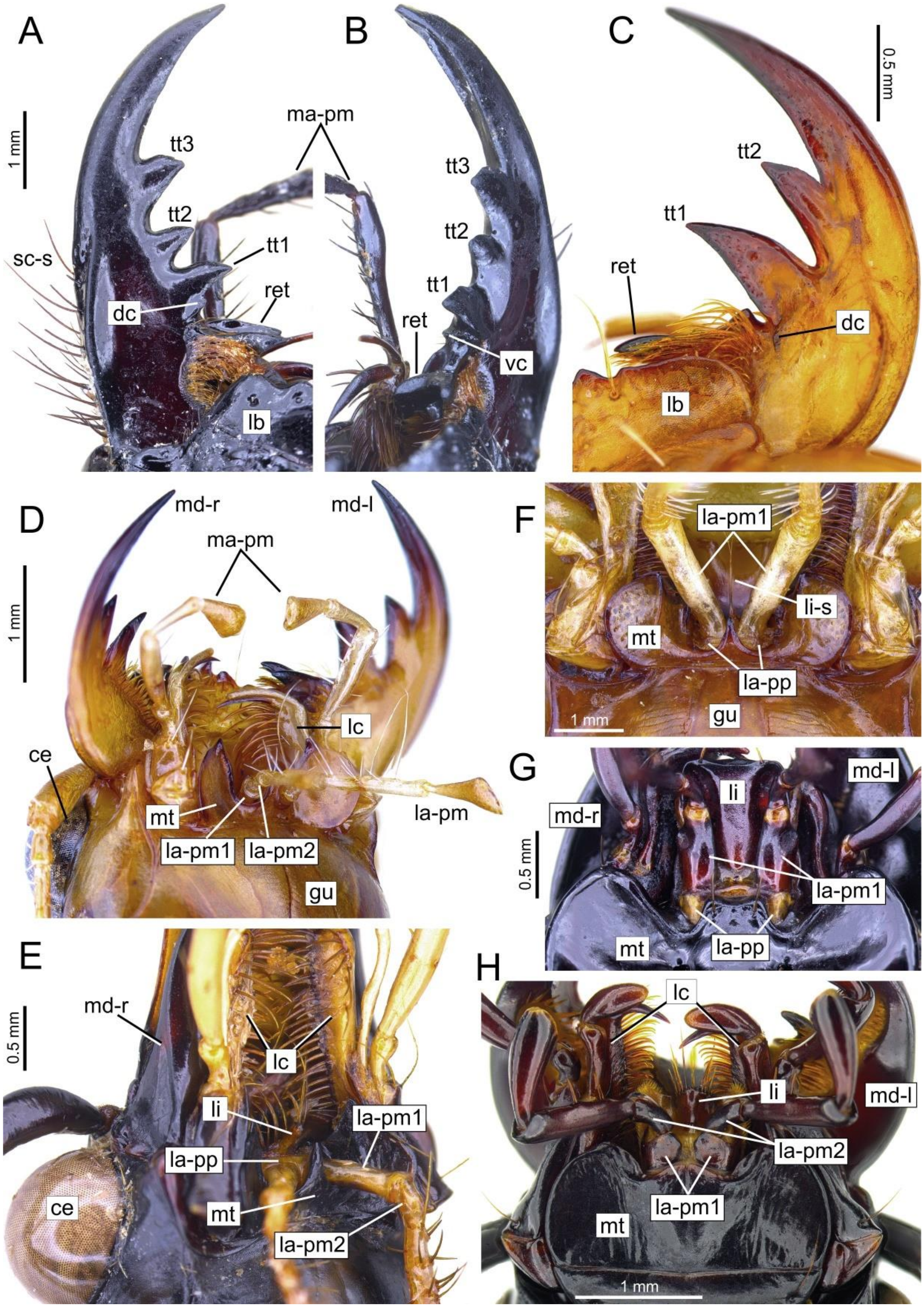

Figure S4. Light microscopic photographs of mandibles (A–C) and ventral side of head (D–H) of *Mantica horni* (Cicindelidae: Manticorini) (A,B), *Platychile pallida* (Cicindelidae: Manticorini) (C,D), *Oxycheila chaudoiri* (Cicindelidae: Oxycheiliniini) (E), *Phaeoxantha klugii* (Cicindelidae: Megacephalini) (F), *Lesticus nepalensis* (Carabidae: Harpalinae) (G), *Carabus ernsti* (Carabidae: Carabinae) (H). Abbreviations: **dc**: dorsal cusp of basal terebral tooth; **gu**: gula; **la-pm**: labial palps; **la-pm1, pm2**: labial palpomeres 1 resp. 2; **la-pp**: labial palpiger; **lb**: labrum; **lc**: lacinia; **li**: ligula; **li-s**: apical setae of ligula; **ma-pm**: maxillary palps; **md-l, -r**: left resp. right mandible; **mt**: mentum; **ret**: retinaculum; **sc-s**: scrobal setae; **tt1, tt2, tt3**: terebral teeth 1–3; **vc**: ventral cusp of basal terebral tooth.
