# Supplementary figures and images for: MicroCT data provide evidence correcting the previous misidentification of an Eocene amber beetle (Coleoptera, Cicindelidae) as an extant species
Source: Sci Rep. 2023 Sep 7;13:14743. doi: 10.1038/s41598-023-39158-7 (PMC10484930; doi:10.1038/s41598-023-39158-7)

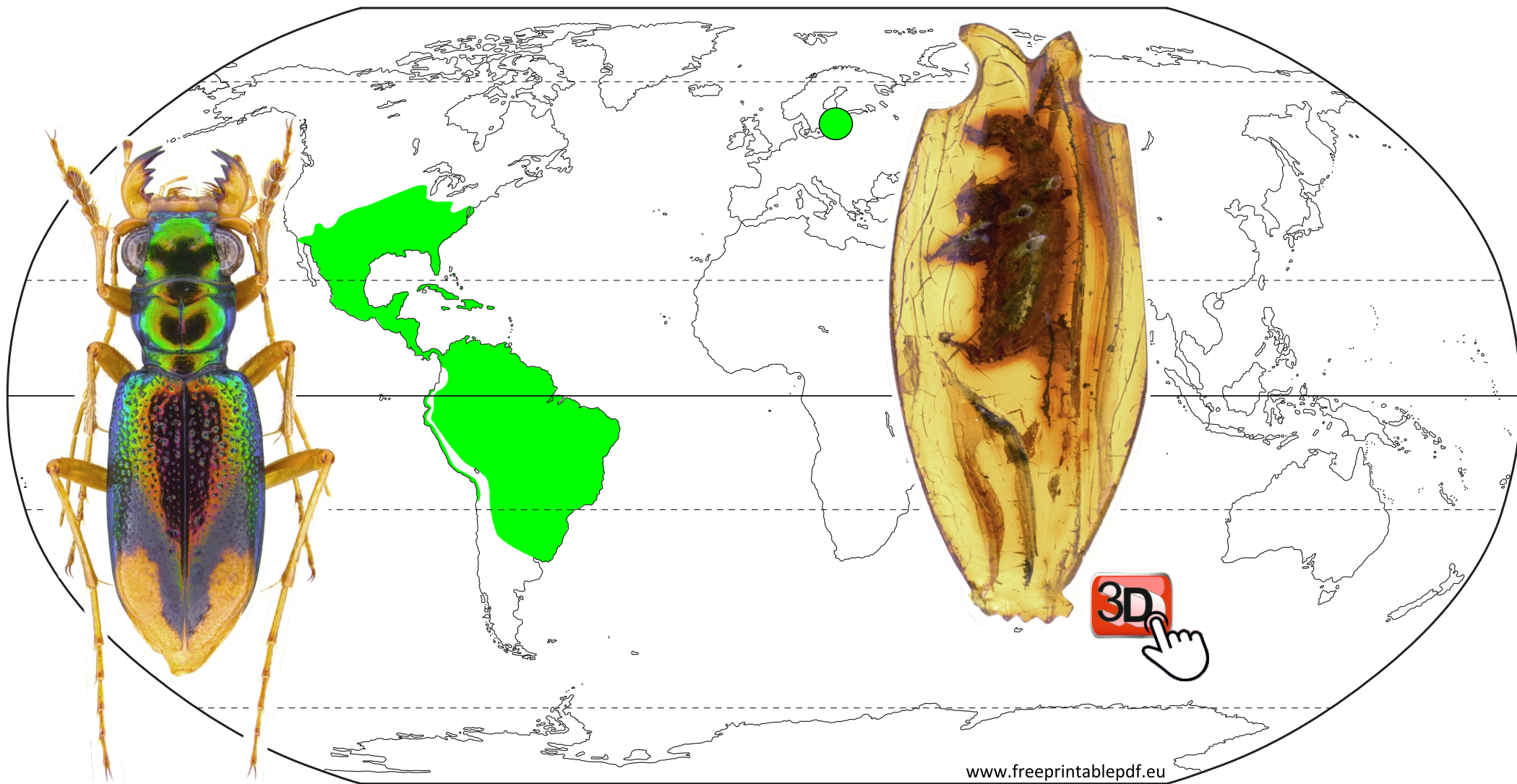

Supplement: Supplementary file 2 — Supplementary Figure 1. [file 41598_2023_39158_MOESM2_ESM.pdf]

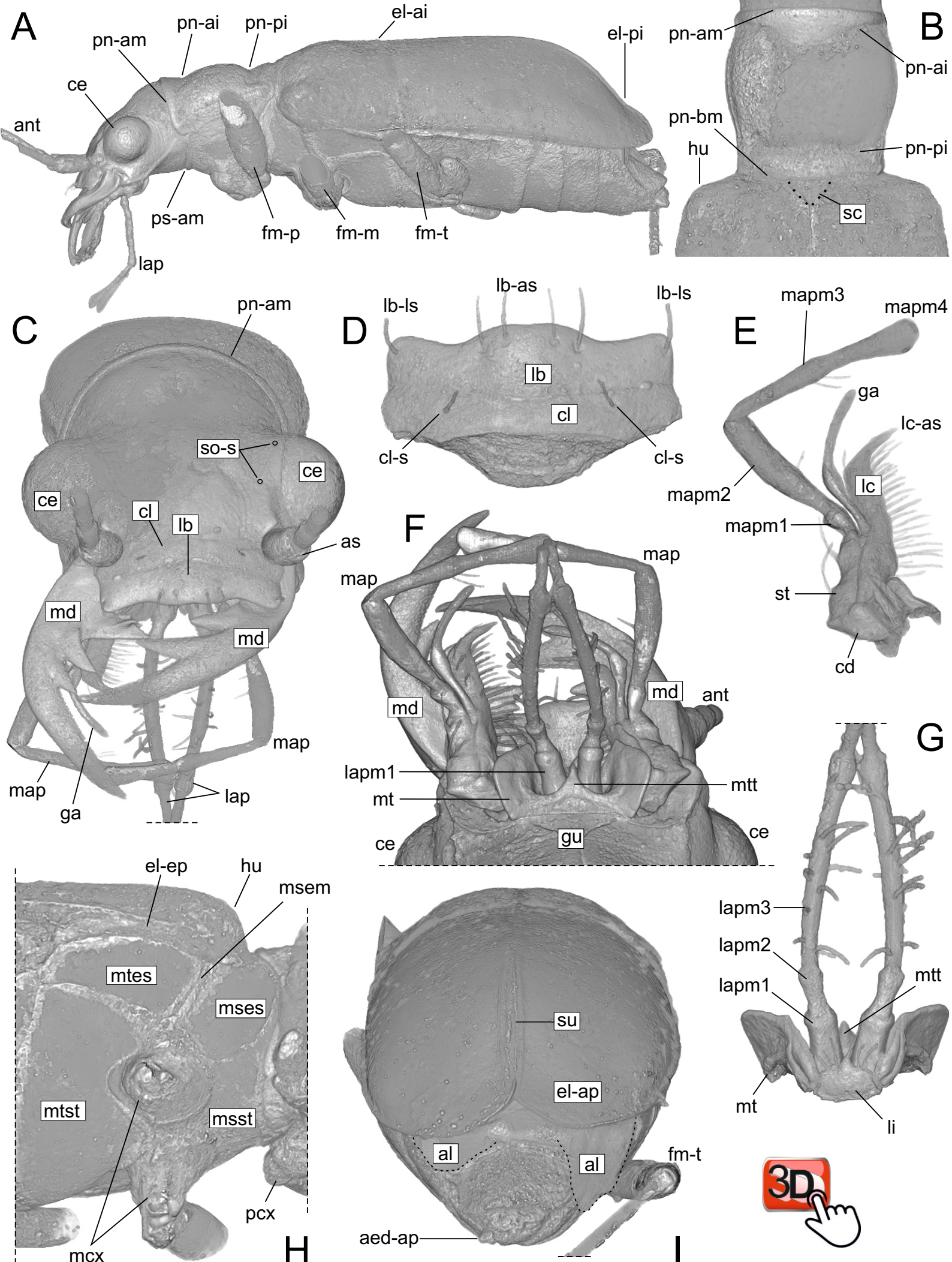

Supplement: Supplementary file 3 — Supplementary Figure 2. [file 41598_2023_39158_MOESM3_ESM.pdf]

**A**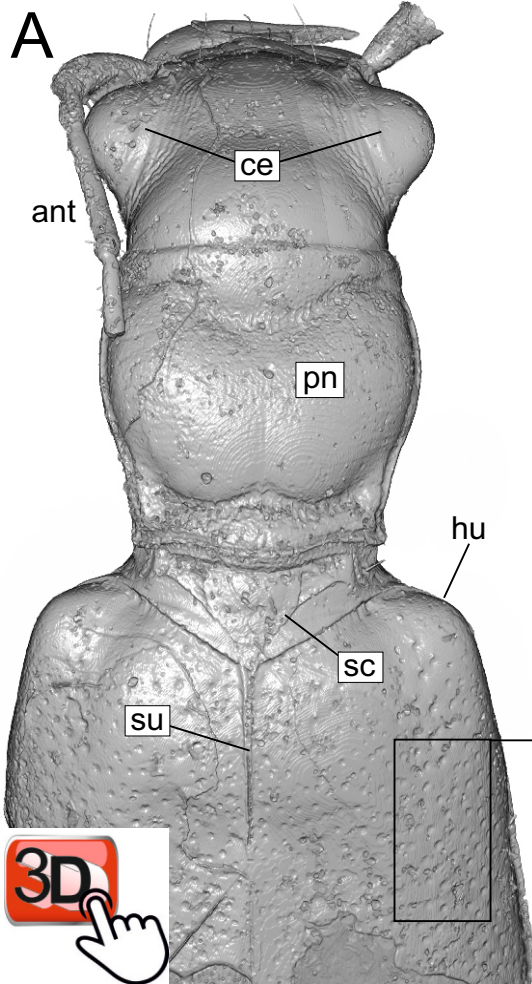**B**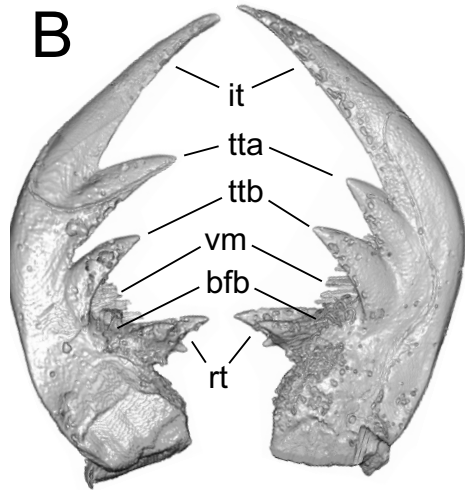**C**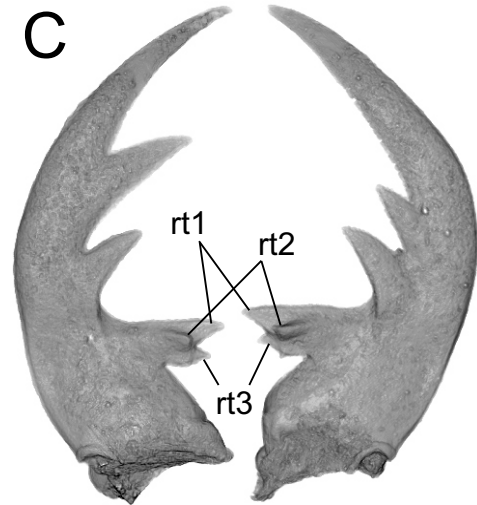**A'**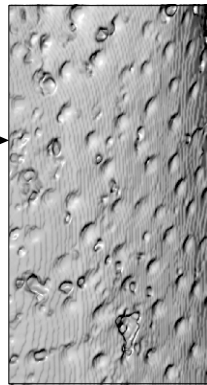**D**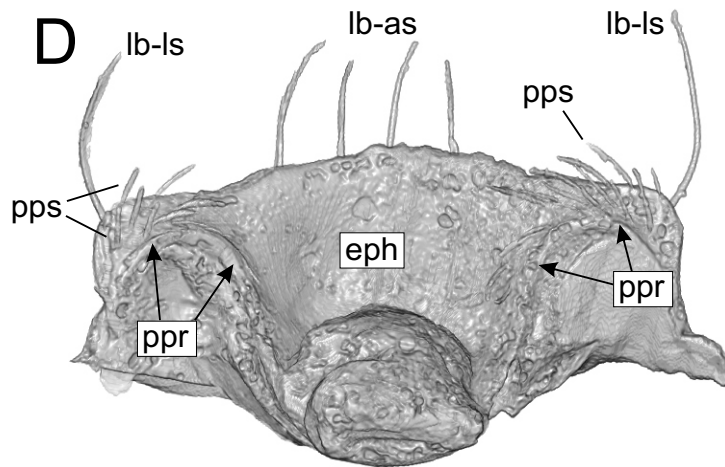

Supplement: Supplementary file 4 — Supplementary Figure 3. [file 41598_2023_39158_MOESM4_ESM.pdf]
